# Examination and Identification of distinct and shared proteomic profiles linked to Alzheimer's Disease biomarkers across multiple proteomic platforms

**DOI:** 10.1002/alz70856_106888

**Published:** 2026-01-08

**Authors:** Hubert Leo, Joshua L Gills, Omonigho M Bubu, The ARIC Neurocognitive Biomarker Workgroup

**Affiliations:** ^1^ New York University Langone Health, New York City, NY, USA; ^2^ NYU Grossman School of Medicine, New York, NY, USA

## Abstract

**Background:**

Plasma biomarkers like Aβ42/Aβ40, *p*‐tau isoforms, and NfL are crucial for assessing Alzheimer's disease (AD) pathology. Advanced platforms such as SomaScan 11k, Olink 5k, and ALAMAR (NULISA‐based) enable precise protein measurement. We examined associations between Alamar's NULISA proteins related to AD (ATN & I) with SomaScan and Olink proteins and conducted pathway enrichment analysis to identify proteomic profiles linked to AD biomarkers.

**Method:**

This cross‐sectional study used plasma samples from 116 ARIC cohort participants (age 74.3 ± 4.7 years, 88.8% cognitively normal). ATN & I biomarkers included: A (Aβ proteinopathy) – Aβ‐42, Aβ‐40, and Aβ‐42/40; T (phosphorylated and secreted AD tau) ‐ *p*‐tau217, *p*‐tau181, *p*‐tau231 and their ratios with Aβ‐42; N (neuropil injury) – NfL and Total Tau; and I (inflammation) – GFAP. Biomarkers were quantified using the ALAMAR NULISA platform and compared to SomaScan 11k and Olink 5k data. Cross‐platform correlations and pathway enrichment analyses (KEGG library) were conducted. Bonferroni correction adjusted for multiple comparisons, and analyses were adjusted for age, sex, APOE ε4 status, and glomerular filtration rate.

**Result:**

There were 3,418 proteins (92 with unique IDs) across SomaScan, Olink, and NULISA platforms. Aβ42 had the most intersecting proteins (268), NEFL had 146, and GFAP had 57. Between Alamar and Olink, Aβ40 had 456 significantly correlated proteins, NEFL had 379, and Aβ42 had 359 after Bonferroni adjustment (*p* < .00001). Between Alamar and SomaScan, Aβ40 had 408 and Aβ42 had 351 significantly correlated proteins (*p* < .00001). In Olink pathway enrichment, Aβ42/40 had the most correlated pathways, while in SomaScan, Ptau231 had the most. Common pathways included cytokine‐cytokine interaction, insulin signaling, MAPK signaling, cell adhesion, and endocytosis.

**Conclusion:**

Our analysis identified shared proteomic profiles associated with AD biomarkers across multiple proteomic platforms. The Alamar ATN & I plasma biomarkers demonstrated moderate to strong correlations with Olink 5k and Soma 11k proteins subserving muscular integrity, inflammation, kidney function, cell membrane integrity, fatty acid degradation. Cytokine‐cytokines and axonal pathways were among the highest correlated pathways among the ATN & I biomarkers across Olink and Soma platforms. Future investigations should examine tissue specific pathways in older individuals across the AD continuum.

